# Identification of *Streptococcus suis* Meningitis by Direct Triplex Real-Time PCR, Burkina Faso

**DOI:** 10.3201/eid2609.200203

**Published:** 2020-09

**Authors:** Mahamoudou Ouattara, Mamadou Tamboura, Dinanibe Kambire, Mahamoudou Sanou, Kalifa Ouattara, Malika Congo, Adama Kaboré, Soufiane Sanou, Elie Kabré, Sable Sharpley, Theresa Tran, Stephanie Schwartz, Soumeya Ouangraoua, Abdoul-salam Ouedraogo, Lassana Sangaré, Rasmata Ouedraogo-Traore, Cynthia G. Whitney, Bernard Beall

**Affiliations:** Centers for Disease Control and Prevention, Atlanta, Georgia, USA (M. Ouattara, S. Sharpley, T. Tran, S. Schwartz, C.G. Whitney, B. Beall);; Centre Hospitalier Universitaire Pédiatrique Charles de Gaulle, Ouagadougou, Burkina Faso (M. Tamboura, D. Kambire, M. Sanou, R. Ouedraogo-Traore);; Centre Hospitalier Universitaire Yalgado Ouédraogo, Ouagadougou (K. Ouattara, M. Congo, L. Sangaré);; Centre Hospitalier Universitaire Sanon-Sourô, Bobo-Dioulasso, Burkina Faso (A. Kaboré, S. Sanou, A-s, Ouedraogo);; Centre-Muraz, Bobo-Dioulasso (E. Kabré, S. Ouangraoua)

**Keywords:** Streptococcus suis, meningitis, direct real-time PCR, Streptococcus agalactiae, Streptococcus pyogenes, zoonotic diseases, streptococci, bacteria, meningitis/encephalitis, infection, Burkina Faso, zoonoses

## Abstract

Meningitis confirmation in Burkina Faso uses PCR for detecting *Streptococcus pneumoniae*, *Neisseria meningitidis*, or *Hemophilus influenzae*. We identified 38 cases of meningitis among 590 that were PCR-positive for 3 nonpneumococcal streptococcal pathogens, including 21 cases of *Streptococcus suis.* Among the country’s 13 regions, 10 had *S. suis–*positive cases.

*Streptococcus suis* is a commensal organism of the upper respiratory tract of pigs that can occasionally cause severe invasive infections in these animals (*1*,*2*). The bacterium also can infect humans who have close contact with pigs or pork products, leading to serious diseases such as meningitis, endocarditis, and sepsis (*3*). *S. suis* can survive in dust, manure, and pig carcasses for days or even weeks under optimal conditions; therefore, the working environments in slaughterhouses and farms can be a source of human infection (*3*,*4*). Meningitis is the most common clinical manifestation of *S. suis* infection in humans and has an estimated case-fatality rate of 3% (*2*). Whereas this zoonotic pathogen is among the leading causes of adult meningitis in some countries of Southeast Asia (*5*), its incidence in Africa is largely unknown. We report 21 retrospectively confirmed cases of *S. suis* meningitis in Burkina Faso.

## The Study

Burkina Faso conducts nationwide case-based surveillance for bacterial meningitis. Cerebrospinal fluid (CSF) samples collected from patients with suspected meningitis are sent to 1 of 5 national laboratories for confirmation by culture and real-time PCR testing (*6*). Because of multiple challenges encountered by the laboratories in isolating bacteria from CSF, the confirmation of meningitis cases relies heavily on molecular detection (*6*). The real-time PCR assay currently used at the national laboratories only detects *Streptococcus pneumoniae*, *Neisseria meningitidis*, and *Haemophilus influenzae*. Between 2015 and 2018, the national laboratories tested 7,174 CSF samples from patients with suspected meningitis nationwide. Of these, 4,930 (68.7%) were PCR-negative for *S. pneumoniae*, *N. meningitidis*, and *H. influenzae*. To further investigate suspected meningitis cases that were PCR-negative after testing for the 3 pathogens, a subset of specimens was selected for additional screening based on leukocytes counts >50 cells/mm^3^. In total, 912 PCR-negative specimens were available for the study; among these, 590 fit the selection criteria. The specimens were retested by using a triplex direct real-time PCR that was designed for the simultaneous detection of *Streptococcus agalactiae* (group B *Streptococcus* [GBS]), *S. pyogenes* (group A *Streptococcus* [GAS]), and *S. suis*. The nucleotide sequences and final concentrations of the primers and probes used in this assay (Table 1) were identical to those used in singleplex real-time PCR methods (*7*–*10*), with the exception that FAM was replaced by HEX as the reporter dye for *cfb*-specific probes for the GBS target and by Cy5 as the reporter dye for *spy*-specific probes for the GAS target. Each reaction was prepared in a final volume of 25 µL, including 1 µL of each primer and probe, 2 µL of CSF as DNA template, 12.5 µL of PerfeCta MultiPlex qPCR ToughMix mastermix (QuantaBio, https://www.quantabio.com), and 1.5 µL of PCR-grade water. The thermal profile for the real-time PCR runs was 1 cycle of 55°C for 5 min, 1 cycle of 95°C for 10 min, then 40 cycles of 95°C for 15 s and 60°C for 1 min. All specimens were tested by real-time PCR for the presence of the human ribonuclease P (RNaseP) gene to check for the presence of inhibitors. A specimen was considered positive if the cycle threshold (C_t_) for 1 of the bacterial targets was <35 and was considered negative if the C_t_ was >40 for all bacterial targets with C_t_ <35 for RNaseP. If C_t_ was between 35 and 40 for the bacterial targets and <35 for RNaseP, the specimen is retested; if the result was reproducible, it was considered positive. The lower limits of detection (LLDs) were determined as previously described (*11*). The LLD of the direct triplex assay (3 CFU/µL for *S. suis*, 28 CFU/µL for GBS, and 26 CFU/µL for GAS) were compared with the LLD of the triplex assay conducted on DNA extracted from the same serial dilutions (2 CFU/µL for *S. suis*, 5 CFU/µL for GBS, and 3 CFU/µL for GAS).

**Table 1:** Sequences and concentrations of primers and probes used to detect *Streptococcus suis*, *S. agalactiae*, and *S. pyogenes*, Burkina Faso*

Target gene	Forward primer, 5¢ ® 3¢	Reverse primer, 5¢ ® 3¢	Probe, 5¢ ® 3¢	Concentration of oligo per reaction, nM†
*cfb* (*7*), GBS	GGGAACAGATTATGAAAAACCG	AAGGCTTCTACACGACTACCAA	AGACTTCATTGCGTGCCAACCCTGAGAC 5¢-HEX; 3¢-BHQ1	200/200/200
*fbpS* (*8*), *S. suis*	TCCRATRCTGCTCTGCCATT	TGATAGTAGAAGTCCAGCARACT	AATAGCCC”T”GAAAAMCAGCCACWYTTTGARA 5¢-FAM; 3¢-SpC6; “T” = BHQ1	200/200/100
*spy* (*9*), GAS	GCACTCGCTACTATTTCTTACCTCAA	GTCACAATGTCTTGGAAACCAGTAAT	CCGCAAC”T”CATCAAGGATTTCGTTACCA 5¢-Cy-5; 3¢- SpC6; “T” = BHQ2	300/300/100
RNaseP (*10*)	AGATTTGGACCTGCGAGCG	GAGCGGCTGTCCCACAAGT	TTCTGACCTGAAGGCTCTGCGCG 5¢-FAM; 3¢-BHQ1	400/400/100

*GAS, group A *Streptococcus* (*Streptococcus pyogenes*); GBS, group A *Streptococcus* (*S. agalactiae*); RNaseP, human ribonuclease P. †Concentration of forward primer/reverse primer/probe.

Among the specimens tested, 21 were positive for *S. suis*, 13 for GAS, and 4 for GBS (Table 2). Of the 21 *S. suis*–positive case-patients, 1 was 4 years of age, 3 were 5–14 years of age, 2 were 15–29 years of age, 9 were 30–49 years of age, and 6 were >50 years of age; 16 (76.2%) were male and 5 (23.8%) were female. Two (9.5%) patients died, 14 recovered, and the outcome for 5 was unknown. Eight (61.5%) of the GAS-positive case-patients were <5 years of age and 5 were 5–14 years of age; 1 (7.7%) patient died, 5 recovered, and the outcome for 7 was unknown. All the GBS-positive case-patients were <6 months of age and all recovered. Median time from symptom onset to CSF collection was 2 days (range 1–10 days) for all the patients.

**Table 2:** Characteristics of patients with meningitis caused by *Streptococcus suis*, *S. pyogenes*, and *S. agalactiae*, Burkina Faso, 2015–2018*

Characteristics	Patients infected, no. (%)			All patients, no. (%), n = 590
	*S. suis*, n = 21	*S. pyogenes*, n = 13	*S. agalactiae*, n = 4	
Age, y				
<5	1 (4.8)	8 (61.5)	4 (100)	307 (52)
5–14	3 (14.3)	5 (38.5)	0	168 (28.5)
15–29	2 (9.5)	0	0	64 (10.8)
30–49	9 (42.8)	0	0	37 (6.3)
>50	6 (28.6)	0	0	14 (2.4)
Sex				
M	16 (76.2)	9 (69.2)	2 (50)	332 (56.3)
F	5 (23.8)	4 (30.8)	2 (50)	258 (43.7)
Year CSF collected				
2015	5 (23.8)	1 (7.7)	0	87 (14.7)
2016	2 (9.5)	2 (15.4)	0	104 (17.6)
2017	6 (28.6)	3 (23.1)	2 (50)	195 (33.1)
2018	8 (38.1)	7 (53.8)	2 (50)	204 (34.6)
Median leukocytes, cells/mm^3^ (range)	400 (51–6,250)	1,000 (53–8,000)	1,100 (800–2,400)	920 (50–8,000)
Median C_t_ (range)	26.1 (18.13–35.52)	25.33 (14.78–34.71)	23.47 (17.24−24.23)	25.33 (14.78–35.52)

*CSF, cerebrospinal fluid; C_t_, cycle threshold.

*S. suis*–positive cases were found among specimens from rural districts in 10/13 regions of the country with 28% (6/21) from the Centre-Est Region and 19% (4/21) from the Plateau Central Region. All adults with *S. suis* were farmers. Because of the retrospective detection of the etiology, the extent of any direct exposure of the *S. suis*–positive patients to pigs or pork products was unknown and attempts to collect additional information from the patients or their families were unsuccessful. 

Small-scale pig farming is a major economic activity in most parts of Burkina Faso. Although porcine-related occupations, undercooked pork consumption, and exposure to pigs have been identified as major risk factors for *S. suis* infection (*5*), its occurrence in a wide range of animal species have been reported (*2*).

DNA extracted from the *S. suis*–positive specimens were used for a multiplex conventional PCR for serotype detection (*12*). All the specimens were serotype 2 or 1/2 because the method used here could not clearly differentiate serotypes 2 and 1/2. Bacterial isolates from these cases were not available for further characterization. Serotype 2 is believed to cause 74%–95% of human cases of *S. suis* infections reported worldwide, but serotype 1/2 has not been reported to cause disease in humans (*1*,*2*).

## Conclusions

Limited data are available regarding the incidence of streptococcal infections in Africa, and the disease burden likely is underestimated, especially for disease caused by *S. suis*. Two reported studies conducted in Togo identified 16 human cases of *S. suis* meningitis during 2010–2015 caused by serotype 2 (*13*,*14*). Another recent study in Madagascar described 2 human cases of *S. suis* meningitis, also caused by serotype 2 (*15*).

Our study reports 21 cases of *S. suis* meningitis from 2015–2018 in Burkina Faso. *S. suis* infections more commonly are observed in adult males and are directly correlated with occupational exposure to pigs or pork products (*2*,*13*,*15*). All adult cases reported here were farmers, although their exposure to pigs or pork was unknown. The presence of children among the patients in this study and in previous studies (*13*) indicates that older children also should be considered at risk. Finding 21 cases of this outbreak-prone zoonotic pathogen in this study raises concern about the incidence of *S. suis* disease in Burkina Faso and in other parts of Africa where pigs are raised. Prospective surveillance for *S. suis* could help identify farming communities where measures to prevent disease and its potential socioeconomic damages are needed.
